# Supplementary figures and images for: Exploiting Single-Cell Quantitative Data to Map Genetic Variants Having Probabilistic Effects
Source: PLoS Genet. 2016 Aug 1;12(8):e1006213. doi: 10.1371/journal.pgen.1006213 (PMC4968810; doi:10.1371/journal.pgen.1006213)

■ BY                      ■ RM                      ■ BY-*DOT6*<sup>RM</sup>

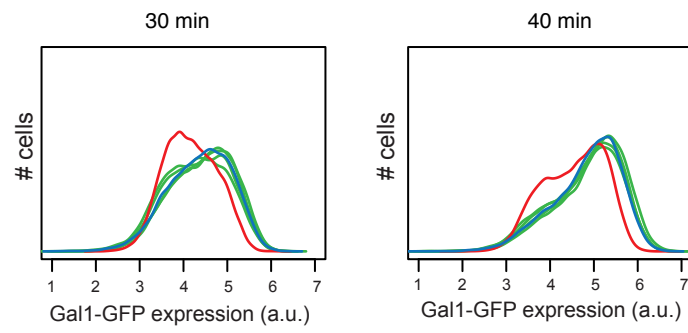

Supplementary Figure 1

Supplement: S1 Fig — Strains GY1566 (BY), GY1567 (RM) and GY1604, GY1605, GY1606, GY1607 (BY-DOT6RM) were cultivated in raffinose 2% and were shifted to a medium containing Raffinose 2% and Galactose 0.5%. After the indicated time, cultures were fixed with paraformaldehyde and analysed by flow cytometry. Histograms correspond to the fluorescent values obtained on cells gated for cell-size (see methods). (PDF) [file pgen.1006213.s001.pdf]

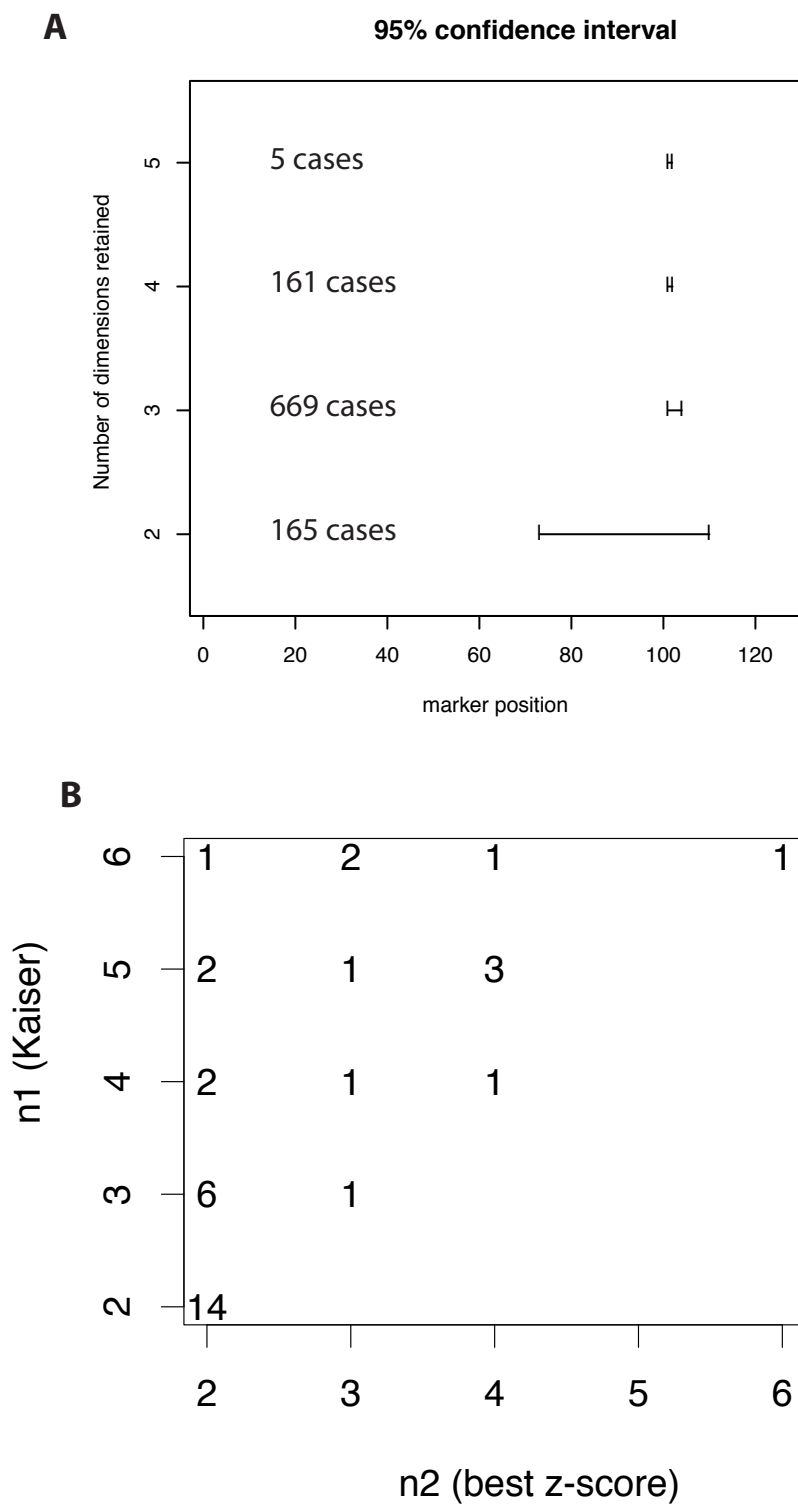

Supporting Figure S2

Supplement: S2 Fig — A) Confidence interval of scPTL location. An example of confidence intervals obtained by bootstrap is shown. In this case, we used the data from 90 individuals shown in Fig 4 (simulations with noise strength η = 0.2). We generated 1,000 bootstrapped samples by randomly choosing individuals, with replacement, and we applied scPTL mapping to each sample. In each case, we recorded the peak position of the scPTL (x-axis) and the dimensionality of the phenotypic space that was retained (y-axis). Lower and upper segment boundaries correspond to the 2.5th and 97.5th percentiles of the observed positions for each dimensionality. B) Dimensionalities retained for the yeast morphological traits. Let n1 be the Kaiser’s based number of dimensions retained after MDS, and n2 the Z-score based number of dimensions retained for linkage. The figure shows the distribution of these values for yeast morphological traits where multi-dimensional scPTL outperformed the one-dimension only. On this plot, the number printed at position x = n2, y = n1 is the number of morphological traits for which the corresponding (n1,n2) values were chosen. (PDF) [file pgen.1006213.s002.pdf]
