# Supplementary material for: Exploiting Single-Cell Quantitative Data to Map Genetic Variants Having Probabilistic Effects
Source: PLoS Genet. 2016 Aug 1;12(8):e1006213. doi: 10.1371/journal.pgen.1006213 (PMC4968810; doi:10.1371/journal.pgen.1006213)
Supplement: S2 Table — (DOCX) [file pgen.1006213.s004.docx]

**Table S2A. Strains used in this study.**

| Name | General Background | Specific genotypes | Source |
| --- | --- | --- | --- |
| BY4716 | S288c | *MATalpha lys2∆0* | *Brachmann et al. [1]* |
| YEF1946 | RM11-1a | *MATa leu2∆0 ura3∆0 ho∆::KanMX amn1-A1103T* | *E. Foss* |
| BY4724 | S288c | *MATa lys2∆0 ura3∆0* | *Brachmann et al. [1]* |
| GY1561 | RM11-1a | *MATa leu2∆0 ura3∆0 ho∆::loxP amn1-A1103T* | *this study* |
| GY221 | S288c | *MATalpha lys2∆0 HIS3:Pgal1-yEGFP3-NatMX:HIS3* | *this study* |
| GY225 | RM11-1a | *MATa leu2∆0 ura3∆0 ho∆::KanMX amn1-A1103T HIS3:Pgal1-yEGFP3-NatMX:HIS3* | *this study* |
| GY689 | RM11-1a | *MATalpha* *leu2∆0 ura3∆0 ho::KanMX amn1-A1103T* | *Abraham et al. [2]* |
| GY739 | RM11-1a | *MATa* *leu2∆0 ura3∆0 ho∆::loxP-KlLEU2-loxP* | *this study* |
| GY744 | RM11-1a | *MATa* *leu2∆0 ura3∆0 ho∆::loxP* | *this study* |
| GY1561 | RM11-1a | *MATa* *leu2∆0 ura3∆0 ho∆::loxP amn1-A1103T* | *this study* |
| GY1566 | S288c | *MATa lys2∆0 ura3∆0 HIS3:Pgal1-GFP_PEST_-NatMX:HIS3* | *this study* |
| GY1567 | RM11-1a | *MATa leu2∆0 ura3∆0 ho∆::loxP amn1-A1103T HIS3:Pgal1-GFP_PEST_-NatMX:HIS3* | *this study* |
| GY1604 | S288c | *MATa lys2∆0 ura3∆0 HIS3:Pgal1-GFP_PEST_-NatMX:HIS3 DOT6^RM^* | *this study* |
| GY1605 | S288c | *MATa lys2∆0 ura3∆0 HIS3:Pgal1-GFP_PEST_-NatMX:HIS3 DOT6^RM^* | *this study* |
| GY1606 | S288c | *MATa lys2∆0 ura3∆0 HIS3:Pgal1-GFP_PEST_-NatMX:HIS3 DOT6^RM^* | *this study* |
| GY1607 | S288c | *MATa lys2∆0 ura3∆0 HIS3:Pgal1-GFP_PEST_-NatMX:HIS3 DOT6^RM^* | *this study* |

**Table S2B. Primers used in this study.**

| ID | 5'- sequence -3' |
| --- | --- |
| 1A23 | CCGGATCCGGGACATTCTAGAGTCGAGATCTTG |
| 1A24 | CCGGATCCCGACTCTTTTCTTCTAAC |
| 1E75 | TACTGGTTGAAACAAATCAGTGCCGGTAACGCTTTTTGTATCTTGAGATGGagctgaagcttcgtacgc |
| 1E76 | TTGAATTGTACTACCGCTGGGCGTTATTAGGTGTGAAACCACGAAAAGTTCgcataggccactagtggatc |
| 1I27 | ttctactaaactaaaccacccccttggttagaagaaaagagtcgggatccGCAGATTGTACTGAGAGTGC |
| 1I28 | gcgcgcaattaaccctcactaaagggaacaaaagctggagctccggctagGCGGCCGCggtgcctaatgagtgagcta |
| 1I42 | agactggaccatcaccaattggag |
| 1I92 | caagtttgaaggtgatacct |
| 1I93 | acgtggactccaacgtcaaagggcgaaaaaccgtctatcagggcgatggcCCTAGGatattaccctgttatccctagc |
| 1J33 | cgcgttctaacgacaatatgtccatatggtgcactctcagtacaatctgcTGTAAAGAGCCCCATTATC |
| 1K87 | tctttcattatgtgagagtttaaaaaccagaaactacatcatcgaaaaagGCCTCGACAAGACGGAATCAGAAA |
| 1K88 | cgcccaatacgcaaaccgcctctccccgcgcgttggccgattcattaatgGTGGCAAATGTGAGGAAGGAAGG |

1. Brachmann CB, Davies A, Cost GJ, Caputo E, Li J, Hieter P, et al. Designer deletion strains derived from Saccharomyces cerevisiae S288C: a useful set of strains and plasmids for PCR-mediated gene disruption and other applications. Yeast. 1998;14: 115–32.

2. Abraham AL, Nagarajan M, Veyrieras JB, Bottin H, Steinmetz LM, Yvert G. Genetic modifiers of chromatin acetylation antagonize the reprogramming of epi-polymorphisms. PLoS Genet. 2012;8: e1002958. doi:10.1371/journal.pgen.1002958
