# Supplementary material for: Exploiting Single-Cell Quantitative Data to Map Genetic Variants Having Probabilistic Effects
Source: PLoS Genet. 2016 Aug 1;12(8):e1006213. doi: 10.1371/journal.pgen.1006213 (PMC4968810; doi:10.1371/journal.pgen.1006213)
Supplement: S1 Methods — (DOCX) [file pgen.1006213.s005.docx]

**Scanning genomes for single-cell Probabilistic Trait Loci**

Florent Chuffart, Magali Richard, Daniel Jost, Claire Burny, Hélène Duplus-Bottin, Yoshikazu Ohya and Gaël Yvert

Supplementary Methods

Description of key steps of scPTL mapping

(R implementation)

Note: The implementation of the methods is fully available as an open source R package called *ptlmapper*: (https://github.com/fchuffar/ptlmapper).

**Calculation of the Kantorovich distance**.

Let x_1_ and x_2_ be the vectors of single-cell trait values for individuals 1 and 2, respectively. We built two histograms *h1* and *h2* of single cell trait values with a common *breaks* attribute:

breaks = seq(min, max, by = nb_bin-1)

h1 = hist(x1, plot = FALSE, breaks = breaks)

h2 = hist(x2, plot = FALSE, breaks = breaks)

with *min* (resp. *max)* being the global minimal (resp. the maximal) value of the single cell trait over all individuals. The number of bins (*nb_bin*) was set to 100 (similar results were obtained at various binning precisions). The distance was then computed using the code:

bin_size = h1$breaks[2] - h1$breaks[1];

KD = bin_size^2*sum(abs(cumsum(h1$density - h2$density))).

**Multi-dimensional Scaling**

Multidimensional scaling was done using function cmdscale() from the stats package. The number of informative dimensions to keep was determined by calling cmdscale a first time with an arbitrary value for the parameter k (here k = 2) and then interrogating how many of the resulting eigenvalues exceeded expectation from a uniform distribution (i.e. if the data has no structure, Kaiser criterion). This was done as follows:

# Compute the number of significant eigen values of multi-dimensional scaling

get_nb_eig_sign = function(pheno_KD) {

mds = cmdscale(pheno_KD, k = 2, eig = TRUE)

mds_eig = mds$eig[mds$eig > 0]

nb_eig_sign = sum(mds_eig/sum(mds_eig) >= 1/length(mds$eig))

return(nb_eig_sign)

}

The output of this function was the resulting number of dimensions nb_dim. If it was lower than 2, we set it to 2. After this, cmdscale was called a second time with k = nb_dim, the desired number of dimensions. This was done as follows:

nb_dim = max(2, get_nb_eig_sign(pheno_KD))

mds = cmdscale(pheno_KD, k = nb_dim, eig = TRUE)

data = data.frame(mds$points)

**Canonical Analysis**

Linear discriminant analysis was done using a custom function seqWilk() that we derived from the candisc() function of the candisc package. The interest of seqWilk() is to extract the W score that we use as test statistics.

############################################################

# A function that returns useful values that are initially #

# embedded in the output of the candisc() function. #

############################################################

seqWilks = function (eig, p, df.h, df.e) {

p.full = length(eig)

result = matrix(0, p.full, 4)

m = df.e + df.h - ( p.full + df.h + 1)/2

for (i in seq(p.full)) {

test = prod(1/(1 + eig[i:p.full])) # this is the Lambda score that we

# use as the test statistics

p = p.full + 1 - i

q = df.h + 1 - i

s = p^2 + q^2 - 5

if (s > 0) {

s = sqrt(((p * q)^2 - 4)/s)

} else {

s = 1

}

df1 = p * q

df2 = m * s - (p * q)/2 + 1

result[i,] = c(test, ((test^(-1/s) - 1) * (df2/df1)),

df1, df2)

}

result = cbind(result, pf(result[,2], result[,3],

result[,4], lower.tail = FALSE))

colnames(result) = c("LR test stat", "approx F",

"num Df", "den Df", "Pr(> F)")

rownames(result) = 1:p.full

return(result)

}

#############################

# Then the actual analysis #

#############################

# Some formatting of the data...

data$allele = as.factor(all)

data = data[!is.na(data$allele),]

# Building the statistical model according to colnames of object data

model_formula = paste("cbind(", paste(names(data)[-length(names(data))],

collapse=", "), ") ~ allele")

# ...followed by the analysis itself

mod = lm(model_formula, data=data)

can = candisc(mod, data=data)

p = can$rank

eig = can$eigenvalues[1:p]

df.h = can$dfh

df.e = can$dfe

tests = seqWilks(eig, p, df.h, df.e)

return(list(W=tests[1,5], can=can, tests=tests))

**Trait heritability in the multi-dimensional phenotypic space**:

Let points be the data matrix giving the coordinates of each sample in the phenotypic space, with the first 3 rows being the replicate samples from one parental strain, rows 4 to 6 the replicate samples from the other parental strain, and rows greater than 7 the samples of the segregant strains (one sample per segregant). Genetic heritability H was computed as follows:

# environmental variance

var_e_p1 = sum(diag(cov(points[1:3,]))) # from first parent

var_e_p2 = sum(diag(cov(points[4:6,]))) # from second parent

var_e = mean(c(var_e_p1, var_e_p2))

# total variance

var_t = sum(diag(cov(points[7:nrow(points),])))

# heritability

H = (var_t - var_e) / var_t
